# Image Sensing and Processing with Convolutional Neural Networks

**DOI:** 10.3390/s22103612

**Published:** 2022-05-10

**Authors:** Sonya Coleman, Dermot Kerr, Yunzhou Zhang

**Affiliations:** 1 School of Computing, Engineering and Intelligent Systems, Ulster University, Londonderry BT48 7JL, UK; d.kerr@ulster.ac.uk; 2 College of Information Science and Engineering, Northeastern University, Shenyang 110819, China; zhangyunzhou@ise.neu.edu.cn

Convolutional neural networks are a class of deep neural networks that leverage spatial information, and they are therefore well suited to classifying images for a range of applications. These networks use an ad hoc architecture inspired by our understanding of processing within the visual cortex. Convolutional neural networks (or CNNs) provide an interesting method for representing and processing image information and form a link between general feed-forward neural networks and adaptive filters. Two-dimensional CNNs are formed by one or more layers of two-dimensional filters, with possible non-linear activation functions and/or down-sampling. CNNs possess key properties of translation invariance and spatially local connections (receptive fields). Given this, deep learning using convolutional neural networks (CNNs) has quickly become the state of the art for challenging computer vision applications.

Image quality is critical for many applications. CNNs have a key role to play in directly dealing with low-quality images or in image enhancement applications. Tchendjou et al. [1] presented a new objective method incorporating a CNN for the estimation of visual perceiving quality without the need for a reference image or assumptions on the image quality. Wang et al. [2] explored the effect of geometric disturbance corresponding to attitude jitter using a GAN to explore the usefulness for jitter detection, revealing the enormous potential of GAN-based methods for the analysis of attitude jitter from remote sensing images. Han et al. [3] proposed a deep supervised residual dense network, which uses residual dense blocks to enhance features along with an encoder and decoder to reduce the differences between the features for underwater degraded images. Xiao et al. [4] focused on blur detection as an image segmentation problem where a multi-scale dilated convolutional neural network (MSDU-net) extracts features with dilated convolutions and a U-shape architecture fuses the different-scale features, supporting the image segmentation task. Yang et al. [5] proposed a novel deeply recursive low- and high-frequency fusing network for single-image super-resolution (SISR) tasks which adopts the structure of parallel branches with a focus on reducing computational and memory resources.

CNNs can play a leading role in environmental applications. For example, pollution in the form of litter in the natural environment is one of the great challenges of our times. Cordova et al. [6] developed an automated litter detection system that can help assess waste occurrences in the environment. A comparative study involving state-of-the-art CNN architectures highlights the role CNNs can play to support this. Similarly, Wei et al. [7] developed models for predicting the wind speed and wave height near the coasts of ports during typhoon periods, where gated recurrent unit (GRU) neural networks and convolutional neural networks (CNNs) were combined and adopted to formulate the typhoon-induced wind and wave height prediction models. Wu et al. [8] targeted the detection of specific crop types from crowdsourced road-view photos and clearly demonstrated the superior accuracy of this approach. Xu et al. [9] presented an accurate and robust detection of road damage that is essential for public transportation safety, and Chou et al. [10] developed a smart dredging construction site system using automated techniques to automate the audit work at the control point, which manages trucks in river dredging areas.

Healthcare is an important application area that AI and CNNs, in particular, can have an impact on. Specifically, the role of 5G-IoT plays a crucial part in e-health applications, and to this end, Anand et al. [11] proposed a new deep learning model to detect malware attacks based on a CNN. In contrast, Barros et al. [12] presented a hybrid model to classify lung ultrasound videos captured by convex transducers to diagnose COVID-19 with an average accuracy of 93% and sensitivity of 97%. The Clock Drawing Test (CDT) is a rapid, inexpensive, and popular screening tool for cognitive functions. Park et al. [13] presented a mobile phone application, mCDT, and suggested a novel, automatic, and qualitative scoring method and deep learning that provides the ability to differentiate dementia disease. Alsamadony et al. [14] applied DCNNs to improve the quality of rock CT images and reduce exposure times by more than 60% simultaneously. The approach is applicable to any computed tomography technology. Ankita et al. [15] presented an approach in which convolutional layers are combined with long short-term memory (LSTM) for human activity recognition (HAR); providing an accuracy of 97.89%, this has applications in assistive living and healthcare.

Robotics is an important application area for CNNs, and to help robots grasp specific objects in multi-object scenes, the powerful feature extraction capabilities of CNNs have been proposed. Different from anchor-based grasp detection algorithms, Li et al. [16] successfully developed a keypoint-based scheme demonstrating that a robot can grasp the target in single-object and multi-object scenes with overall success rates of 94% and 87%, respectively.

This Special Issue provides a forum for high-quality peer-reviewed papers that broaden the awareness and understanding of recent CNN developments, applications of CNNs for computer vision tasks, and associated developments in CNN architectures, processing components, connective structures, and learning mechanisms, and in dealing with CNN constraints in respect to data preparation and training.
